# Bone Age Delay in X-linked Hypophosphatemia

**DOI:** 10.1210/jendso/bvaf184

**Published:** 2025-11-20

**Authors:** Julio Soto, Rucha Anant Patki, Lauren J Ehrlich, Rachana Borkar, Alba León, Elizabeth A Olear, Cicero T Silva, Thomas O Carpenter

**Affiliations:** Department of Pediatrics (Endocrinology), Yale School of Medicine, New Haven, CT 06520, USA; Las Higueras Hospital, Talcahuano 4270918, Chile; Department of Pediatrics, Faculty of Medicine, University of Concepcion, Concepcion 4070106, Chile; Department of Pediatrics (Endocrinology), Yale School of Medicine, New Haven, CT 06520, USA; Department of Radiology and Biomedical Imaging, Yale University School of Medicine, New Haven, CT 06519, USA; Yale-New Haven Health-Bridgeport Hospital, Bridgeport, CT 06610, USA; National School of Public Health, “Héctor Abad Gómez,” University of Antioquia, Medellín 050015, Colombia; Department of Pediatrics (Endocrinology), Yale School of Medicine, New Haven, CT 06520, USA; Department of Radiology and Biomedical Imaging, Yale University School of Medicine, New Haven, CT 06519, USA; Department of Pediatrics (Endocrinology), Yale School of Medicine, New Haven, CT 06520, USA

**Keywords:** X-linked hypophosphatemia, skeletal maturation, bone age, predicted adult height

## Abstract

**Context:**

Bone age (BA) assessment and prediction of adult height (AdHt) has not been well studied in children with X-linked hypophosphatemia (XLH).

**Objective:**

To assess BA and its utility in height prediction in children with XLH.

**Design:**

Retrospective, cross-sectional, and longitudinal assessments of BA using 2 standard methods in children with XLH. Mean values were used to calculate predicted adult height (PAH), which was compared to final or near-final AdHt in patients who were at or near the end of growth.

**Setting:**

Academic medical center.

**Patients:**

Fifty-six children with XLH.

**Intervention:**

None.

**Main Outcome Measures:**

BA, PAH.

**Results:**

Initial radiographs demonstrated BA delay (chronologic age–BA) of 1.2 ± 1.0 (mean ± SD) years in males and 0.4 ± 1.0 years in females (greater delay in males, *P* < .05). Fifty-eight percent of males and 21% of females were delayed 1 to 2 years; 11% of males and 9% of females were delayed more than 2 years. For 4 males with no prior orthopedic surgeries, mean AdHt was 171.2 ± 5.3 cm; PAH was 176.3 (±11.7) cm using Bayley-Pinneau methods and 173.0 ± 6.8 cm per Tanner-Whitehouse methods. For 15 females without prior orthopedic surgeries, AdHt was 155.9 ± 5.2 cm; PAH was 156.0 ± 6.8 cm (Bayley-Pinneau) or 161.6 ± 4.2 (Tanner-Whitehouse, which differed from AdHt, *P* < .005).

**Conclusion:**

BA is delayed in children with XLH but more strikingly so in males. Height predictions were within a range typically used in healthy children (±2 inches). The Bayley-Pinneau method appears to modestly overestimate AdHt in males, whereas Tanner-Whitehouse overestimates AdHt in females.

X-linked hypophosphatemia (XLH), due to loss-of-function variants of *PHEX* with resultant excess circulating fibroblast growth factor 23 [1], is characterized by renal phosphate-wasting and chronic hypophosphatemia. Accompanying defects in vitamin D metabolism result in low circulating levels of 1,25-dihydroxy vitamin D [2, 3]. Children with XLH are clinically affected with impaired skeletal mineralization presenting as rickets and characteristic short stature [4]. *PHEX* encodes an endopeptidase highly expressed in bone (osteoblasts, osteocytes) and teeth (odontoblasts) and a variety of other phenotypic findings are often present, including recurrent dental abscesses and craniosynostosis [5].

At birth, length and weight in children with XLH fall within normal centiles, but height velocity begins to decrease near 1 year of age [6]. Later, when XLH patients undergo pubertal growth spurts, there is not a complete recovery of the prior loss in growth, usually resulting in a significant compromise in adult height (AdHt) [6, 7]. Parental concern about height outcomes, as in the management of children without XLH, often leads to the assessment of skeletal age in order to predict AdHt; however, bone age (BA) determination by commonly employed methods has not been systematically evaluated in children with XLH. Typical approaches to this end include the methods of Bayley-Pinneau (BP) [8], Roche et al [9], and Tanner-Whitehouse (TW) [10]. More recently, computer-based algorithms have automated this process but currently are not in widespread use. All methods are based on estimates of skeletal maturity, manifest by configuration, and degree of mineralization of carpal bones, as well as growth plate regions of the distal radius and ulna, metacarpal, and phalangeal bones. Published standards allow for a comparison to age-related healthy children [8-10].

A delay in the appearance of epiphyseal bone centers has been described in rickets, which may affect the interpretation of skeletal age using these methods. The epiphysial growth plate is an active site of endochondral ossification, which determines the final length of bones [11]. Indeed, *PHEX*, the gene in which pathogenic variants occur in XLH, is strongly expressed by chondrocytes [5], suggesting that its loss of function may directly disturb long bone growth in XLH patients [11]. Despite these concerns, characterization of skeletal maturation during growth in XLH, and the utility of BA as a predictor of AdHt in affected children has not been systematically evaluated. Our primary objective was to assess skeletal maturation in XLH patients using the methods commonly employed in most clinical settings. Our secondary objective was to assess the accuracy of AdHt prediction using BA in patients with XLH.

## Materials and Methods

This retrospective study was conducted at Yale University School of Medicine and included 226 BA radiographs from 56 patients followed at the Yale Pediatric Metabolic Bone clinics, obtained between April 2012 and October 2022. The primary inclusion criteria were a clinical diagnosis of XLH and the availability of radiographs of the left hand/wrist during their course. Patients under 4 years of age were excluded due to limited standards for BA determination. All radiographs had been previously performed locally. The majority were obtained for the evaluation of rickets in clinical trials and a long-term monitoring program (sponsored by Ultragenyx, accessed with permission); 3 patients (all females) had been radiographed for clinical care purposes independent of research protocols. The study was reviewed and approved by the institutional review board at the Yale University School of Medicine.

The primary analysis was performed using the initial X-ray for each patient. Each image was interpreted by 2 radiologists and 2 endocrinologists using 2 commonly applied methods [Greulich and Pyle (GP) and Gilsanz and Ratib (Gil)] [12, 13]. All 4 readers were blinded to the date of the radiograph, associated patient identifiers excepting sex, as well as each other's readings. For each method, the mean of the 4 readings was used as the BA. If a substantial outlier occurred (a reading greater than 1.5 years from the mean), a fifth senior radiologist provided an adjudicated revision of the outlier BA reading, which replaced the outlier and was incorporated into the mean score. Of all 226 BA radiographs, 13 (6%) required adjudication by the fifth reader per the GP method and 15 (7%) BA films per the Gil method, attesting to the substantial agreement across the primary readers (≥97%).

To quantitate the degree of delay, the difference between BA and chronological age (CA) was calculated at the time each radiograph and classified into 3 levels: less than 1 year, greater than 1 and less than 2 years, and more than 2 years. Height ages (the age at which the current height of the patient corresponds to the 50th percentile for height, using the Centers for Disease Control and Prevention height for age charts) [14] were calculated based on measured height at the time of radiographs.

Factors that may influence the degree of delay, such as pubertal status (breast Tanner stage in females and genital appearance in males), obesity [body mass index (BMI)], and disease severity (serum phosphorus and total alkaline phosphatase levels), were assessed at the time of the first radiograph.

Predicted adult height (PAH) was ascertained using suggested methods per each atlas; BP methods were applied to the GP-determined BA readings and TW methods were applied to Gil-determined readings, as per the appendices in their respective atlases [8, 10]. In patients with growth velocities of less than 0.5 cm/year, PAH was assessed by both methods for their proximity to final or near-final AdHt. We performed these analyses using both the initial and final readings for each patient, and a secondary analysis was done excluding all patients who had undergone surgical intervention of the lower extremities.

### Statistical Analysis

Means and SDs were calculated for BA delay and height predictions. Dispersion graphs were performed to evaluate the correlation between the variables of interest, and Spearman correlation coefficients were calculated. Student *t*-tests were applied to determine the central tendency measures for paired and independent samples depending on the direction of comparisons in the total group and by sex. The relative frequency of BA delay and its classification according to severity were calculated. Concordance between the readings obtained by the 2 methods utilized was evaluated through the Bland–Altman limits of agreement analysis, with *P*-values less than .05 considered significant. All analyses were performed using Stata Software version 14 (StataCorp, College Station, TX, USA).

## Results

### Patient Characteristics

Baseline characteristics of the 53 (19 males/34 females) patients are summarized in Table 1. At the time of initial radiographs, 26% of males were overweight and 16% were obese, and 21% of females were overweight and 21% were obese per BMI criteria. The ethnic/racial composition of participants was largely Caucasian (82%) and non-Hispanic (99%). Mean age of the male participants (8.3 years) at the time of initial radiographs was slightly less than that for females (9.6 years). Most had a positive family history of XLH (73.6%) or a known pathogenic variant in *PHEX* (60.3%). Almost all patients (96%) were treated with burosumab at some point. Only 2 patients (both females) were entirely managed with oral phosphate and activated vitamin D products.

**Table 1.** Clinical and demographic characteristics of the subjects

	Males	Females	Total
Age at initial radiograph (yrs)	8.3 ± 3.1	9.6 ± 3.2	9.1 ± 3.2
Range	4.3-13.8	4.7-16.7	4.3-16.7
Age at final radiograph (yrs)	11.9 ± 3.5	13 ± 3.3	12.3 ± 3.4
Range	6-17	6-17	6-17
Sex	19 (36%)	34 (64%)	53
BMI (kg/m^2^) at initial radiograph	18.9 ± 3.9	19.8 ± 3.6	19.5 ± 3.7
z-score	0.8 ± 1.0	0.9 ± 0.8	0.8 ± 0.9
Height (cm) at initial radiograph	119.4 ± 18.3	126.3 ± 16.3	123.8 ± 17.2
z-score	−1.4 ± 1.2	−1.5 ± 1.3	−1.4 ± 1.2
BMI (kg/m^2^) at final radiograph	20.4 ± 4.9	22.3 ± 5.0	21.6 ± 5.0
z-score	0.5 ± 1.2	0.7 ± 0.9	0.6 ± 1.0
Height (cm) at final radiograph	141.8 ± 20.2	143.4 ± 14.5	142.8 ± 16.6
z-score	0.5 ± 1.2	0.7 ± 0.9	0.6 ± 1.0
Tanner stage at initial radiograph	2 ± 1.1	2 ± 1.6	2 ± 1.5
Ethnicity			
Caucasian	17	25	42
African American	1	2	3
Hispanic	1	5	6
Other	0	2	2
+ XLH family history	17	22	39
*PHEX* mutation	12	20	32
Missense	5	9	14
Nonsense	3	4	7
Splice-site	3	6	9
Deletion	1	1	2
Treatment			
Burosumab	19	32	51
Conventional	0	2	2
Age at start of burosumab (years)	6.8 ± 4.2	8.9 ± 4.1	8.1 ± 4.2
Range	1-15	1-17	1-17

No significant differences were observed between male and female subjects for these parameters.

Abbreviations: BMI, body mass index; XLH, X-linked hypophosphatemia.

### Radiographic Assessments

#### Analysis of initial radiographs

**Figure 1.**
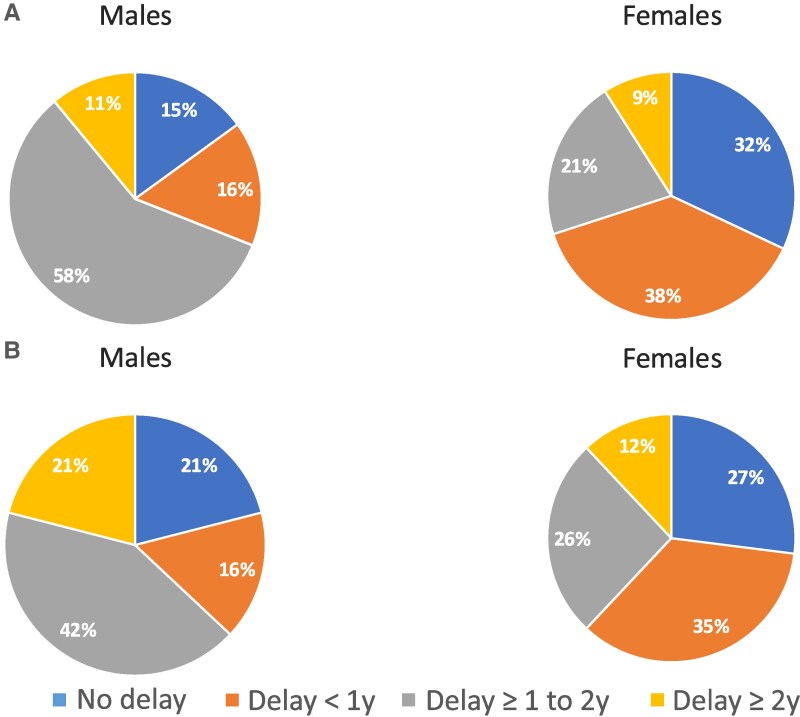
(A) Degree of bone age delay—initial radiographs per Greulich and Pyle method. (B) Degree of bone age delay—initial radiographs per the Gilsanz and Ratib method.

The mean (±SD) BA of the 19 initial radiographs for male patients was 7.1 ± 3.5 years per GP and 7.2 ± 3.9 years per Gil at the concomitant CA of 8.3 ± 3.1 years. Females (34 initial radiographs) had a mean BA of 9.1 ± 3.6 years per GP and 8.9 ± 3.7 years per Gil at the concomitant mean CA of 9.6 ± 3.2 years. A significantly greater BA delay (CA-BA) was observed in males compared to females using GP (1.2 ± 1.0 vs 0.4 ± 1.0, *P* < .05) (Table 2). Applying GP methods, 16 males (84%) were delayed, whereas 23 (68%) of the females were delayed (there was no difference in proportion delayed, *P* = .854). Among males, 58% had a delay between 1 and 2 years and 11% were delayed greater than 2 years, which differed statistically from females using GP (*P* = .036) in whom 21% had a delay between 1 and 2 years and 9% were delayed more than 2 years (Table 2, Fig. 1A). The prevalence of delay and degree of severity was not significantly different when using the Gil methodology (Fig. 1B).

**Table 2.** Bone age delay per Greulich and Pyle

	Males	Females	Total
**All radiographs**	85	129	214
CA (yr)*^a^*	10.1 ± 3.3	11.2 ± 3.0	10.8 ± 3.2
BA (yr)	9.0 ± 3.9	10.8 ± 3.6	10.1 ± 3.8
Height age	8.6 ± 3.4	9.3 ± 2.6	9.0 ± 3.0
BA delay (CA-BA) yr*^b^*	1.0 ± 1.3	0.4 ± 1.2	0.7 ± 1.3
# with any delay (%)*^c^*	68 (80)	83 (64)	151 (71)
BA delay < 1 yr*^d^*	18 (21)	40 (31)	58 (27)
≥ 1 to 2 yr	35 (41)	31 (24)	66 (31)
≥ 2 yr	15 (18)	12 (9)	27 (13)
**Initial radiographs only**	19	34	53
CA (yr)	8.3 ± 3.1	9.6 ± 3.2	9.1 ± 3.2
BA (yr)	7.1 ± 3.5	9.1 ± 3.6	8.4 ± 3.7
Height age	6.8 ± 2.9	7.8 ± 2.7	7.5 ± 2.8
BA delay (CA-BA) yr*^b^*	1.2 ± 1.0	0.4 ± 1.0	0.7 ± 1.1
# with any delay (%)	16 (84)	23 (68)	39 (74)
BA delay < 1 yr*^d^*	3 (16)	13 (38)	16 (30)
≥ 1 to 2 yr	11 (58)	7 (21)	18 (34)
≥ 2 yr	2 (11)	3 (9)	5 (9)

Abbreviations: BA, bone age; CA, chronological age.

^a^Females were older than males when considering the time at which all radiographs were performed (*P* < .05).

^b^BA readings were more severely delayed in males vs females for all and initial radiographs (*P* < .01).

^c^Percentage of delayed males was greater than that of delayed females when considering all radiographs (*P* < .05).

^d^Severity of delay was more pronounced in males for all and initial BA readings (*P* < .05).

Of the 70% of subjects in whom Tanner stages were recorded at the time of the first BA, mean Tanner stage ± SD was 2 ± 1.5 and 55% were Tanner stage 1, with no sex differences (*P* = .163). Furthermore, no differences in the distribution of Tanner stages according to sex were found (*P* = .377). Six patients were postmenarchal at this assessment.

Mean BMI z score was 0.8 ± 0.9 (0.9 ± 0.8 in females and 0.8 ± 1.0 in males, *P* = .629). Regression analysis showed a weak but not statistically significant inverse correlation between BA delay and BMI z-score in the total group [Pearson correlation coefficient (Ro) = −0.23, *P* = .097].

#### Analysis of complete set of radiographs

A total of 214 hand/wrist radiographs were assessed, 85 (40%) from males and 129 (60%) from females in the complete data set (Table 2). Females were older than males (11.2 ± 3.0 vs 10.1 ± 3.3, *P* = .012) when considering the time at which all radiographs were performed. Male patients showed a mean delay of approximately 1 year, similar to the degree of delay seen from the initial radiographs and significantly greater than the delay in females (*P* < .01 by GP and Gil). For the complete set of readings, mean BA in males was 9.0 ± 3.9 years per GP and 9.0 ± 4.0 years per Gil methods at a CA of 10.1 ± 3.3 years. Similarly, females had a mean BA of 10.8 ± 3.6 years per GP and 10.7 ± 3.7 years per Gil at CA of 11.2 ± 3.0 years. A strong concordance was found between the 2 methods [correlation coefficient 0.994 (confidence interval 0.993-0.996)] when considering all radiographs. Of the 85 radiographs from males, 68 (80%) were delayed per GP, whereas for females, 64% were delayed (*P* = .014). For all male films, 41% were delayed 1 to 2 years and 18% were delayed more than 2 years. For all female films, 24% were delayed 1 to 2 years and 9% were delayed more than 2 years (Table 2). BAs were more severely delayed among males when compared with females by GP and Gil (*P* < .05).

### BA, Height Age vs CA

**Figure 2.**
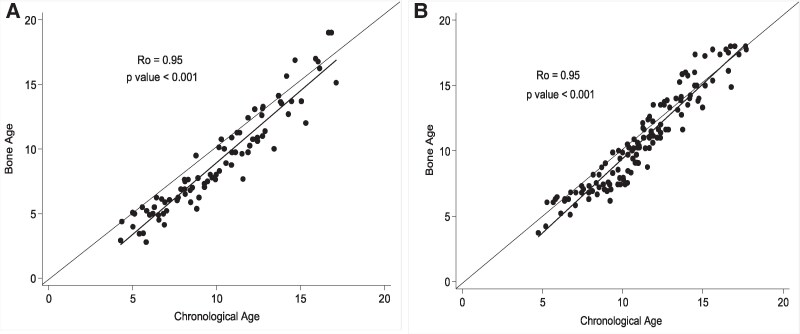
(A) Comparison of bone age vs chronological age for males. (B) Comparison of bone age vs chronological age for females. Data is presented from all radiographs for all patients at each timepoint. Abbreviation: Ro, Spearman correlation coefficient.

**Figure 3.**
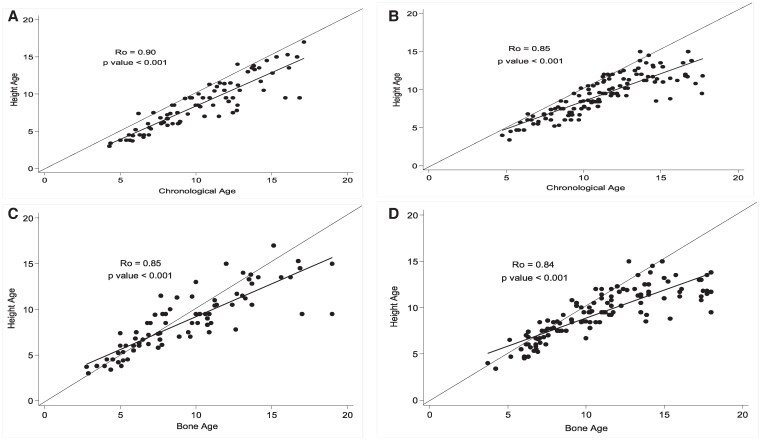
Comparison of height age vs chronological (A, B) and height age vs bone age (C, D) in males (A, C) and females (B, D). Data is presented from all radiographs for all patients. Abbreviation: Ro, Spearman correlation coefficient.

We descriptively assessed longitudinal changes in BA (per GP), height age, and CA (Figs. 2 and 3). BA continually lagged behind the CA in both males (Fig. 2A) and females (Fig. 2B); however, the differences appeared to diminish in mid- to late adolescence (Fig. 2). Height age continually lagged behind CA for both males and females (Fig. 3A and 3B). Height age, although comparable to BA at early years, fell behind BA as patients matured (Fig. 3C and 3D).

### Relationship of BA Delay to Serum Phosphorus and Alkaline Phosphatase Activity

**Figure 4.**
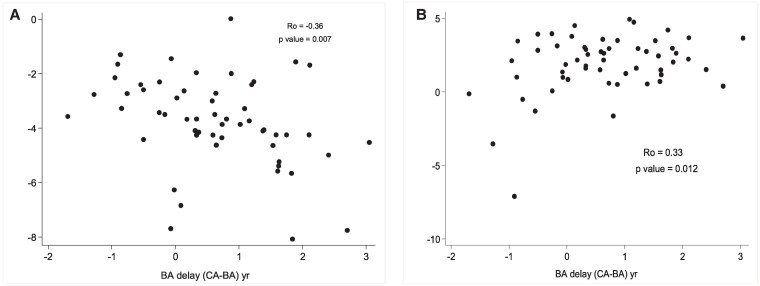
Regression analysis of bone age delay (based on initial radiographs) vs serum phosphorus z score (A) and vs serum alkaline phosphatase activity z score (B). Abbreviation: Ro, Spearman correlation coefficient.

We examined the potential association between biomarkers of disease severity obtained at the time of the initial BA radiograph (Fig. 4). A significant inverse correlation was found between BA delay and serum phosphorus z-score (Ro = −0.36, *P* = .007). Conversely, a significant positive correlation was observed with serum alkaline phosphatase activity (Ro = 0.33, *P* = .012). When examining females and males separately, only the correlation of alkaline phosphatase activity z-score in females remained significant (Ro = 0.49, *P* = .002).

### PAH

PAH was calculated using initial as well as final BAs. PAH obtained using each method was then compared to the final height/near final height (AdHt), which was attained in 5 boys and 20 girls (Table 3). Using initial radiographs, we found no significant difference in mean PAH between the 2 methods, either in males (BP: 171.5 ± 14.7 vs TW: 168.8 ± 11.2, *P* = .752) or in females (BP: 153.6 ± 8.8 vs TW: 158.7 ± 8.1, *P* = .052) (Table 3). Similarly, no differences in PAH were identified between these methods using the final set of radiographs, either in males (BP: 172.3 ± 17.7 vs TW: 171.4 ± 8.8, *P* = .921) or in females (BP: 153.7 ± 9.3 vs TW: 154.5 ± 9.5, *P* = .779), and were more closely matched to AdHt than when using initial radiographs. Application of the TW method overestimated AdHt from the initial radiographs in both males and females but was significant only in females (158.7 ± 8.1 vs 152.9 ± 8.0, *P* = .028).

**Table 3.** Height predictions

		Males (n = 5)	Females (n = 20)
Bayley-Pinneau	Initial PAH	171.5 ± 14.7	153.6 ± 8.8
	Final PAH	172.3 ± 17.7	153.7 ± 9.3
	AdHt	166.4 ± 11.6	152.9 ± 8.0
	*P*-value initial vs final	.883	.972
	*P*-value initial vs AdHt	.174	.794
	*P*-value final vs AdHt	.297	.772
Tanner-Whitehouse	Initial PAH	168.8 ± 11.2	158.7 ± 8.1
	Final PAH	171.4 ± 8.8	154.5 ± 9.5
	AdHt	166.4 ± 11.6	152.9 ± 8.0
	*P*-value initial vs final	.694	.141
	*P*-value initial vs AdHt	.748	.028
	*P*-value final vs AdHt	.465	.568
*P*-value initial PAH (Bayley-Pinneau vs Tanner-Whitehouse)		.752	.052
*P*-value final PAH (Bayley-Pinneau vs Tanner-Whitehouse)		.921	.779

Abbreviations: AdHt, adult height; PAH, predicted adult height.

A subsequent analysis considered only data from patients with no history of orthopedic procedures (Table 4), which may alter height considerably. Mean PAH values using initial radiographs in this subset did not show significant differences between the 2 prediction methods in males (BP: 176.3 ± 11.7 vs TW: 173.0 ± 6.8, *P* = .643), but in females the TW method estimated a significantly higher PAH (BP: 156.0 ± 6.8 vs TW: 161.6 ± 4.2, *P* = .011) (Table 4). Using final radiographs, no significant differences were found between the 2 methods for males (BP: 176.5 ± 17.4 vs TW: 174.3 ± 6.7, *P* = .821) or females (BP: 156.8 ± 6.8 vs TW: 157.6 ± 7.2, *P* = .757). The sole comparison in which the PAH statistically differed from the AdHt was observed for females when using the initial radiograph and TW method (161.6 ± 4.2 vs 155.9 ± 5.2, *P* = .003).

**Table 4.** Height predictions for patients without surgical intervention

		Males (n = 4)	Females (n = 15)
Bayley-Pinneau	Initial PAH	176.3 ± 11.7	156.0 ± 6.8
	Final PAH	176.5 ± 17.4	156.8 ± 6.8
	AdHt	171.2 ± 5.3	155.9 ± 5.2
	*P*-value initial vs final	.985	.750
	*P*-value initial vs AdHt	.457	.964
	*P*-value final vs AdHt	.581	.687
Tanner-Whitehouse	Initial PAH	173.0 ± 6.8	161.6 ± 4.2
	Final PAH	174.3 ± 6.7	157.6 ± 7.2
	AdHt	171.2 ± 5.3	155.9 ± 5.2
	*P*-value initial vs final	.795	.074
	*P*-value initial vs AdHt	.691	.003
	*P*-value final vs AdHt	.495	.465
*P*-value initial PAH (Bayley-Pinneau vs Tanner-Whitehouse)		.643	.011
*P*-value final PAH (Bayley-Pinneau vs Tanner-Whitehouse)		.821	.757

Abbreviations: AdHt, adult height; PAH, predicted adult height.

## Discussion

Short stature is a major clinical feature of XLH [4] that often persists in the face of current therapeutic options [15, 16]. BA is often obtained to assess progressive skeletal maturation in children with short stature and for the prediction of AdHt. Skeletal maturity may be affected by nutrition, metabolic status, socioeconomic status, and environmental factors. Genetic factors and altered hormonal status may also determine skeletal maturity [17, 18]. We describe here the impact of rickets on skeletal maturation, with particular attention to XLH.

Our study was focused on ascertaining BA patterns in children with XLH and assessing the accuracy of height predictions. We found that BA was commonly delayed in patients with XLH, consistent with previous descriptions of prepubertal XLH patients with growth retardation who participated in studies of GH treatment [19, 20]. There was considerable heterogeneity in the degree of delay, despite consistency in some delay across a large proportion of patients. Furthermore, we found that the degree of delay was greater among males than females. Additionally, our data support the use of the Gil method in the XLH population since the results were equivalent to those obtained by GP.

These findings support a possible role of XLH-mediated rickets in the regulation of skeletal maturation. Indeed, mouse models of XLH have shown severe abnormalities in the growth plate of young Hyp mice including reduced chondrocyte proliferation, apoptosis with profound disturbances of chondrocyte hypertrophy, and maturation [11]. Moreover, impairment in hypertrophic chondrocyte apoptosis and expansion of the hypertrophic chondrocyte layer has been described in Hyp mice lacking 1-α-hydroxylase (*Cyp27b1* knockout) and *Fgf23* (heterozygous for *Fgf23* ablation), findings that were not seen in the absence of the *Phex* deletion, suggesting a Phex-dependent, fibroblast growth factor 23-independent role for 1,25 dihydroxy vitamin D on growth plate maturation in XLH [21]. Finally, our study suggests that disease severity may play a role in bone maturation due to the association observed between BA delay and z-scores for serum phosphorus and alkaline phosphatase. On the other hand, as no significant sex differences in BMI or Tanner stages were found, we cannot attribute these variables as an explanation for the differences in the degree of delay between males and females.

To the best of our knowledge, this is the first study to examine the validity of BA as a predictor of AdHt in the XLH population. Height predictions derived from BA assessment in our study were highly correlated with actual AdHt, and their accuracy was within ranges typically used in clinical practice for the non-XLH population. Nevertheless, a modest overestimation of AdHt using TW methods was observed in females; however, height prediction was somewhat overestimated in males using the BP method. Similarly, another study that compared various methods of height prediction in the general population found that the BP method overestimated final height in males [22].

Besides these commonly used methods, automated BA reading techniques have recently been developed for obtaining height predictions, such as BoneXpert® [23]. However, data is lacking regarding the validity of BA and height predictions in children with rickets. In a recent comparison of height prediction methods in healthy children, the BP method was a better predictor of AdHt than TW or BoneXpert [24]. In contrast, among children with chronic endocrinopathies, automated methods showed greater proximity to AdHt than the BP method [25]. Although promising, BoneXpert is not widely available in clinical settings due to costs, and traditional methods remain the most widely used tools for height predictions in practice globally. Comparison with automated methods will be valuable for the future; however, this report adds validity to currently used methods.

As expected, we found that height age lags behind the CA for males and females, in accordance with the short stature seen in XLH patients [6]. In addition, height age was concordant with BA in younger patients; however, it tended to lag behind the BA in late childhood due to an apparent peripubertal acceleration in skeletal maturation. This is in accordance with previous data reporting that most of the height loss in children with XLH occurs prior to puberty and is not regained despite showing a normal growth spurt [6, 7, 26]. Recently reported data indicate a delay in the pubertal growth spurt in patients with XLH in comparison with reference to the Centers for Disease Control and Prevention population, with the former showing a mean growth velocity peak at age 15 in males and 12 in females compared with a peak at age 13 years and 11 years, respectively, in the latter group [27]. These findings are in agreement with the degree of BA delay observed in this study. Indeed, the lesser degree of delay in females with XLH as compared to males is consistent with other subtle differences between the sexes, whereby females may be less severely affected. Features of XLH that have been reported or suggested to differ include height [6] and skeletal phenotype with certain variants [28]. These differences may well relate to the heterozygous state in females, in which the expected expression of the PHEX protein would be normal in one-half of cells in females, whereas no normal PHEX would be expressed in the hemizygous males.

Our study was limited by a small cohort of patients with a condition that variably affects growth and with scant information about final height, particularly in males who were still growing at the time of the analyses, which compels us to interpret these data cautiously. Nonetheless, in light of the available information, height prediction appeared to be generally accurate across both sexes: both methods resulted in a similar prediction of AdHt and were highly correlated whether using initial or final BA readings. A modest overestimation of AdHt in females using initial radiographs by the TW method was found. Despite evidence that burosumab may improve height velocity within the first 3 years of exposure [29], we were not able to assess the impact of burosumab compared to conventional treatment on height prediction since our primary analysis considered the earliest BA radiograph from each patient, at which time very few patients were receiving burosumab. Nevertheless, we infer that the delay does not appear to be influenced by burosumab considering the degree of delay is not substantially different at later BAs, at which time most of the patients had been receiving a variable exposure to burosumab. Further studies are needed with a larger group of patients who have reached final height to confirm our findings and to address the question regarding a potential role of novel therapies on skeletal maturation in XLH. We conclude that there is a systematic delay of BA throughout childhood in children with XLH and that usual methods of AdHt prediction are likely useful clinical tools in this population.

## Data Availability

Datasets generated during and/or analyzed during the current study are not publicly available but are available from the corresponding author upon reasonable request.

## Current Affiliation

The current affiliation of Alba Leon is Biostatistics, Deparlab Research Group, Secretary of Health and Social Inclusion, Government of Antioquia, Medellín 050015, Colombia.
